# Developing a Web-Based Geolocated Directory of HIV Pre-Exposure Prophylaxis-Providing Clinics: The PrEP Locator Protocol and Operating Procedures

**DOI:** 10.2196/publichealth.7902

**Published:** 2017-09-06

**Authors:** Aaron J Siegler, Susan Wirtz, Shannon Weber, Patrick S Sullivan

**Affiliations:** ^1^ Department of Epidemiology Rollins School of Public Health Emory University Atlanta, GA United States; ^2^ University of California, San Francisco San Francisco, CA United States

**Keywords:** HIV prevention, service directory, geolocated database, PrEP

## Abstract

**Background:**

Human immunodeficiency virus (HIV) pre-exposure prophylaxis (PrEP) is highly effective in preventing HIV transmission, yet patients interested in learning more about PrEP or in getting a PrEP prescription may not be able to find local medical providers willing to prescribe PrEP.

**Objective:**

We sought to create a national database of PrEP-providing clinics to allow for patients to have access to a unified, vetted source of PrEP providers in an easily accessible database.

**Methods:**

To develop the protocol and operating procedures for the PrEP Locator, we conducted a series of 7 key informant interviews with experts who had organized PrEP or other HIV service directories. We convened an external advisory committee and a collaborators board to gain expert and community-situated perspectives.

**Results:**

At its public release in September 2016, the database included 1,272 PrEP-providing clinics, including clinics in all 50 states and in Puerto Rico. Web searches, referrals, and outreach to state health departments identified 58 unique lists of PrEP-providing clinics, with 33 from state health departments, 6 from government localities, 2 from professional medical organizations, and 19 from nongovernmental organizations. Out of the 2,420 clinics identified from the lists and Web searches, we removed 798 as duplicate entries, and we determined that 350 were ineligible for listing. The most common reasons for ineligibility were not having the appropriate medical licensure to prescribe PrEP (67/350) or not prescribing PrEP, based on self-report (192/350). Key informant interviews shaped important protocol decisions, such as listing clinics instead of individual clinicians as the primary data element and streamlining data collection to facilitate scalability. We developed a Web interface to provide public access to the data, with geolocated data display, search filter functionality, a webform for public suggestions of new clinics, and a publicly available directory Web tool that can be embedded in websites. In the 6 months following release, preplocator.org and hosting websites had received over 35,000 unique views and 300 clinic additions, and 5 websites had initiated hosting of the widget.

**Conclusions:**

Directories exist for many preventive and treatment services. As new medical applications become available, there will be a corresponding need to develop new directories for service provision. Geolocated directories can assist patients in accessing care and have the potential to increase demand for and access to newer, more efficacious medical interventions. Early choices in the development of service directories have long-lasting impact, because once data collection begins, it can be challenging to reverse course. The PrEP Locator protocol may inform early decisions in the development of future service directories. Additionally, the case study on developing the PrEP Locator demonstrates the importance of formative work in identifying service-specific factors that can guide decisions on directory development.

## Introduction

HIV pre-exposure prophylaxis (PrEP) is highly efficacious in preventing HIV, preventing between 56% and 100% of new infections across several effectiveness studies [1-3]. Additionally, bringing PrEP to scale would have a substantial impact on the overall HIV epidemic, with one mathematical model indicating that scaling PrEP use to 40% of eligible men who have sex with men (MSM) based on US Centers for Disease Control and Prevention (CDC) eligibility criteria would reduce the number of new HIV infections in this group by 33% [4]. Moreover, this intervention is cost-effective for at-risk MSM [5-7]. MSM are not the only group for whom PrEP is appropriate; the CDC estimates that substantial numbers of women (468,000) and injection drug users (115,000) are eligible for PrEP [8]. Given the effectiveness and cost-effectiveness of PrEP, it is an essential public health priority to bring PrEP to scale as rapidly as possible. However, PrEP uptake has been limited, even in the highest-risk groups [9-12].

To better characterize the ways in which PrEP uptake occurs, we have previously specified a PrEP care continuum that includes awareness and willingness, access to health care, the likelihood of receiving a prescription, and adherence and efficacy [13]. PrEP awareness estimates among MSM in the United States vary widely (11%-68%), in part according to region [14-18] but also according to time, as studies with more recent data collection indicate higher awareness estimates [14-18]. Willingness to take PrEP and intention to take PrEP are not synonymous, however, with one study identifying a substantial gap between PrEP willingness and intention [19].

Individuals who are aware of PrEP and intend to initiate it need to find a clinician prepared to prescribe PrEP. Any clinician licensed to prescribe medication can prescribe PrEP, but only 7% of primary care providers in one national survey reported ever prescribing PrEP [20]. Moreover, less than 1% of the 1500 clinicians correctly answered a series of four true-or-false questions regarding PrEP, with “don’t know” being the most commonly selected answer for each question [20]. Encouragingly, however, 22% reported reading CDC clinical practice guidance regarding PrEP. Difficulty in identifying PrEP providers has been described as a “purview paradox”, in which neither specialists (viewing PrEP as needing broad provision and therefore being the domain of primary care doctors) nor primary care doctors (feeling a lack of experience, particularly in prescribing antiretroviral medication) feel that they are the appropriate group to prescribe PrEP [21,22]. Despite the purview paradox, a substantial number of primary and specialist clinicians, found in local lists of PrEP-providing clinics, are willing to prescribe PrEP.

There has previously been no single place for patients to look for a clinician willing to prescribe PrEP. A panoply of local resources have been developed to serve as directories of PrEP clinicians, including clinic lists furnished by states, local health departments, community-based organizations, and even medical provider associations such as the HIV Medicine Association. However, such a fragmented system has a number of limitations. First, accessibility is limited because there is no single place for those interested in PrEP to look; rather, the information is dispersed, and the burden is on the patient to identify the appropriate resource. Second, and related, the ease of accessing and sorting through current lists is mixed. A few directories are location-based lists with accessible, map-based interfaces [23-25], but the majority either are published in list form on the Web or require a telephone call to the relevant agency. Third, the lists have varying quality; some lists have no discernable vetting criteria, and others are not up to date. Fourth, in absence of a single and thorough system to identify where PrEP-providing clinics are located, there is no way to identify pockets where PrEP services are least accessible. Identifying areas of low PrEP service access would allow for targeted recruitment and training of clinicians who could prescribe PrEP. Last, few of the lists identify important services at or features of particular clinics. With a unified national database, it is possible to track and publish searchable lists of services that patients might want to be able to access. For instance, patients might want to be able to identify clinics that prescribe services for patients without insurance or clinics that provide PrEP financial “navigation” services that help patients enroll in manufacturer or state medication assistance or copayment programs [26].

This protocol describes the creation of a national database of PrEP-providing clinics. We conducted developmental planning work, including forming advisory boards and conducting key informant interviews with those experienced in developing HIV and PrEP service directories. We then populated the database by gathering and collating PrEP clinic data from existing resources. We added new data fields, including data to allow for eligibility determination and data that we could present to the public, and conducted data collection to furnish information for these new data fields. While populating the database, we developed an accessible tool for patients to search for clinics in a geolocated fashion, hosted at preplocator.org. To maximize accessibility, we also made the database available through an open-source widget interface that can be added to any website. The goal of this protocol is to document the process used to create a geolocated service directory of PrEP-providing clinics, informing interpretation of the data and serving as a foundation for future efforts to develop PrEP or other health service directories.

## Methods

To develop the protocol and operating procedures for the PrEP Locator, we had several sources of data and advisement, including convening an external advisory committee and a collaborators board and conducting of a series of key informant interviews. The purpose of the External Advisory Committee was to provide guidance regarding the goals of the PrEP Locator project and included representatives from the CDC, community-based organizations, and the activist community. We convened quarterly meetings to receive advice from this group. The purpose of the Collaborators Board was to provide detailed input on implementation of the PrEP Locator and included key community representatives and nongovernmental partners. The Collaborators Board convened monthly at project initiation and biweekly in the months preceding public release of the PrEP Locator, in September 2016.

By conducting a series of key informant interviews, we sought to build on the experiences of those who have previously developed service directory resources. The 7 interviews were with experts who have organized PrEP or other HIV service directories at the local or national level. Participants included representatives from community-based organizations, city and state health departments, and federal HIV prevention efforts. The interviews topics were (1) strategies for identifying and recruiting clinics for inclusion in a database, (2) eligibility criteria for clinics, (3) areas of data collection, (4) methods of managing a database, (5) maintaining and updating clinic data, and (6) lessons learned in running local and national service clinic directories. Because the recommendations and lessons learned were largely mechanistic in nature, the data did not require in-depth textual analysis, with analysis instead via a review of interview notes. We also recorded the interviews, and for areas in which further detail was needed, we reviewed the source material.

## Results

### Advisory Boards

Based on early meetings of the External Advisory Committee and Collaborators Board, we came to several consensus decisions regarding database accessibility and development. First, the database would be easily available for hosting on any site through a simple, location-based widget interface. Second, we settled on an ethos of including as many PrEP-providing clinics as possible by minimizing eligibility criteria. Eligibility criteria would be restricted to either (1) the information necessary for database operation or (2) the information required to allow for determination of appropriate patient access. Operationalizing this ethos meant that eligible clinics only needed (1) a correct address and contact information and (2) verbal or electronic verification that the clinic prescribes PrEP, using state licensure databases to ensure that the clinic has at least one provider with appropriate and up-to-date licensure to prescribe medication such as PrEP. We would not perform more advanced vetting, such as by performing secret shopper calling, determining minimum clinic hours (and even making such hours available), or performing dedicated PrEP scheduling or service navigation. The rationale was that the External Advisory Committee and Collaborators Board were concerned that overly stringent eligibility criteria could exacerbate low numbers of PrEP-providing clinics in resource-poor and rural areas. Third, each collaborating organization agreed to provide specific input within its relative domain of expertise. This feedback covered topics including marketing, graphical design, database design, clinical expertise, and promotional strategy. These areas of feedback substantially informed diverse yet key project components, such as strategies for vetting clinics, the PrEP Locator logo and branding, details of the website graphical interface, and engagement with community partners to ensure reaching those most in need of access to the service.

### Key Informant Interviews

There was substantial consensus across key informant interviews regarding how the PrEP Locator database should identify and recruit clinics, maintain and update clinic data, and manage data. Interview participants recommended that we have a maximally inclusive process, identifying clinics by including all publicly available clinic and clinician lists (governmental and nongovernmental groups included), contacting health departments and medical organizations for unpublished lists, and conducting keyword Web searches state by state.

To update clinic data, participants recommended that we have a webform located on a “front-end” (publicly visible) website and that we also set up regular automatic distribution of this form (eg, biannually) to clinics in the directory along with a request to update any information that has changed. Similarly, informants recommended a front-end accessible webform to add new clinics to the database. To manage webform updates and new clinic entries, participants recommended a “holding pen” system on the database back-end (visible only through rights-based access), allowing database administrators to fact-check information prior to it being added to the publicly available dataset. Regarding database management, a single, cloud-based relational database was recommended, with import and export functionality, an administrative interface to edit data, and rule functions for each database variable or object to ensure data quality.

Interview participants had more divergent views regarding how the PrEP Locator database should determine clinic eligibility criteria. There was a substantial range of eligibility criteria for PrEP clinics across different databases. The minimum threshold used by some was to have the clinics self-complete a brief electronic or paper survey or even a single, opt-in item in a larger clinician survey. One directory used “mystery calling”, a process in which staff would call each clinic and claim to be a patient searching for PrEP. Key informants used the mystery calling system as a point to start discussions with clinics in their database that failed to provide a minimum level of care access. However, after a certain period of attempting to improve outcomes, repeat failures could result in removal of a clinic from the list. Features explored in “mystery” calls included front desk staff indicating awareness of PrEP and knowledge of where to connect the calls or which clinicians at the institution prescribe PrEP, a reasonable wait time for clinic visits, and reasonable access to appointment scheduling. All PrEP clinic directories using more rigorous eligibility criteria served urban areas with a high density of PrEP clinics. At the state level and among local efforts with less funding, directories used substantially fewer eligibility criteria. For the vast majority of PrEP clinic lists, the only inclusion criterion was self-report that a clinic prescribed PrEP.

Across interviews, key informants agreed that certain basic data fields should be included in the database: clinic address, clinic name, clinic contact information (telephone number, email address, and website), and provider name. There was less agreement regarding other domains of data collection, including types of PrEP services offered, hours of operation, and more extensive clinic vetting criteria. Some noted personal experiences with building databases with too many fields, noting that with each additional field, the time required for initial collection and subsequent updating might substantially increase. Some interviewees told stories of users who relied on incorrectly listed hours for a clinic, resulting in delays in obtaining appropriate care. However, other key informants thought that data fields such as hours, PrEP services for those without insurance, or language availability would provide an important service to database users. PrEP services generally require a clinician appointment, so one participant noted that any errors in clinic hours would be unlikely to negatively impact users. Others suggested the possibility of including additional data fields but not making their completion a requirement for a clinic to be included in the public display of PrEP-providing clinics.

Interviewees preferred listing clinics, rather than individual clinicians within each clinic, as a database entry. Some directory developers had initially listed individual clinicians but found it logistically infeasible to maintain an up-to-date list. Individual providers not only frequently changed clinic affiliation but also often shared their times across different offices or clinics. Therefore, it was considered too resource intensive to maintain an individual list of clinicians. Furthermore, interviewees indicated that an individual provider-based system was problematic because it would clutter any geolocated interface, resulting in an unwieldy interface for potential users.

Based on this input from advisory boards and key informants, we developed a protocol for the PrEP Locator database. For areas without consensus, we returned to seek further input from our advisory boards, with the project’s principal investigator being responsible for final decisions. In decisions regarding database development and feature inclusion, we weighed resource availability and the costs and benefits to end users.

### PrEP Locator Protocol

#### Purpose

Emory University, supported by the MAC AIDS Fund, developed a national directory of clinics providing HIV PrEP. The national directory of PrEP-providing clinics, along with its public-facing website, preplocator.org, is termed the PrEP Locator. The PrEP Locator’s goal is to serve as a common repository for information regarding clinics that prescribe PrEP. The directory is an open resource for those who are managing existing directories, allowing them to share their resources in a common format so that patients can access a national, integrated PrEP clinic location service that includes both public and private-practice PrEP providers. The database is easily accessible at preplocator.org or through a public-facing widget that allows distribution through existing websites and mobile apps.

#### Procedures

##### Eligibility criteria

The PrEP Locator database includes both public and private health care clinics that are willing to prescribe PrEP. For all clinics listed in the database, Emory staff verify that at least one provider has current licensure to prescribe medication such as PrEP based on publicly available licensure information posted by state medical boards. Appropriate professional licensure includes an M.D., D.O., N.P., or P.A. degree, which would allow for a clinician to prescribe PrEP. To identify a provider at each clinic to allow for state licensure searching, we rely on either publicly available information or the clinic’s self-report. Publicly available information comes from organizational websites that list providers as part of their staff and from inference for eponymous clinics (eg, for Dr. Jane Doe’s Clinic, we consider Jane Doe to be the provider). Organizations may also self-report to Emory staff that a provider at the organization can prescribe PrEP. Self-report data come from email, webforms, or telephone calls. Regardless of source, staff then enter clinician names into state licensure databases as search criteria to determine appropriate and up-to-date licensure. Pharmacies prescribing PrEP through collaborative drug therapy agreements are eligible for inclusion, with vetting conducted based on the name and credentials of the clinician overseeing the agreement. Prior to being included in the database, pharmacies must submit relevant information to allow for standard eligibility determination. Due to privacy concerns for providers and to organizations’ internal vetting, Planned Parenthood clinics and other public clinics known for offering birth control prescriptions are exempt from this vetting process. For all other organizations, if we are unable to verify a licensed provider on staff, the organization is not eligible for inclusion in the directory.

Another eligibility criterion is that clinics must have complete data for four variables: *clinic name*, *address* (street address, county and/or city, state, and zip code), and *telephone number* and *provider name* (to verify licensure). If these data fields are not available from the data used to populate the database, Emory staff contact the clinic or provider electronically or with a telephone call. If unable to establish contact, Emory staff make connections through use of Web-based keyword searches to correct potentially incorrect contact information. If Emory staff are still unable to make contact after 3-7 additional telephone calls, they consider the clinic or provider not eligible for database inclusion. Eligible clinics must also serve the general public, so some student health clinics and Veterans Administration clinics are not included in the database. The call script used for clinic eligibility determination and additional data collection, when available, is in Multimedia Appendix 1.

##### Procedure for Database Population

Development of the PrEP Locator dataset, conducted from October 2015 to September 2016, used Web searches, referrals, and outreach to state health departments to identify PrEP-providing clinics. Emory staff entered all PrEP-providing clinics into a single database. For the majority of clinics, information was not available in database-friendly electronic format, therefore requiring manual data entry by Emory staff. During the process of data entry, staff removed duplicate entries. To develop the final dataset, we then made a case-by-case eligibility determination for each clinic. For clinics with insufficient data available from the original data source to determine eligibility, we either called the clinic or used information available on the Web, based on a clinic’s website, to collect sufficient data to make a determination.

To identify sources of PrEP-providing clinics, we first identified state health department lists by using Web searches and by referral from our advisory boards. Next, Emory staff conducted keyword searches by state, between January 2016 and August 2016, for each of the following terms: “(PrEP OR pre-exposure prophylaxis) providers (state)”, “(PrEP OR pre-exposure prophylaxis) HIV providers (state)”, “clinics that prescribe (PrEP OR pre-exposure prophylaxis) (state)”, and “(PrEP OR pre-exposure prophylaxis) HIV prevention (state)”. For all states without publicly available clinic lists, Emory staff contacted the state health department director or the state director of HIV prevention to request lists, if available. Our searches further identified other governmental sources that list PrEP-providing clinics, such as city, county, and regional directories. We included clinician lists from professional organizations, the HIV Medicine Association and the American Academy of HIV Medicine, and we identified nongovernmental lists, such as lists of community-based organizations, social media groups (eg, PrEP Facts), and foundations, through similar means and included these as well. Last, we included individual PrEP-providing clinics identified during the Web search process.

##### Database Release

At database release in September 2016, we had identified a total of 58 unique lists of PrEP-providing clinics, including 33 from state health departments, 6 from government localities (city or county), 2 from professional medical organizations, and 19 from nongovernmental organizations. Prior to screening and deduplication, PrEP-providing clinics across these lists totaled 2346, and we added an additional 74 clinics based on Web searches.

**Figure 1:**
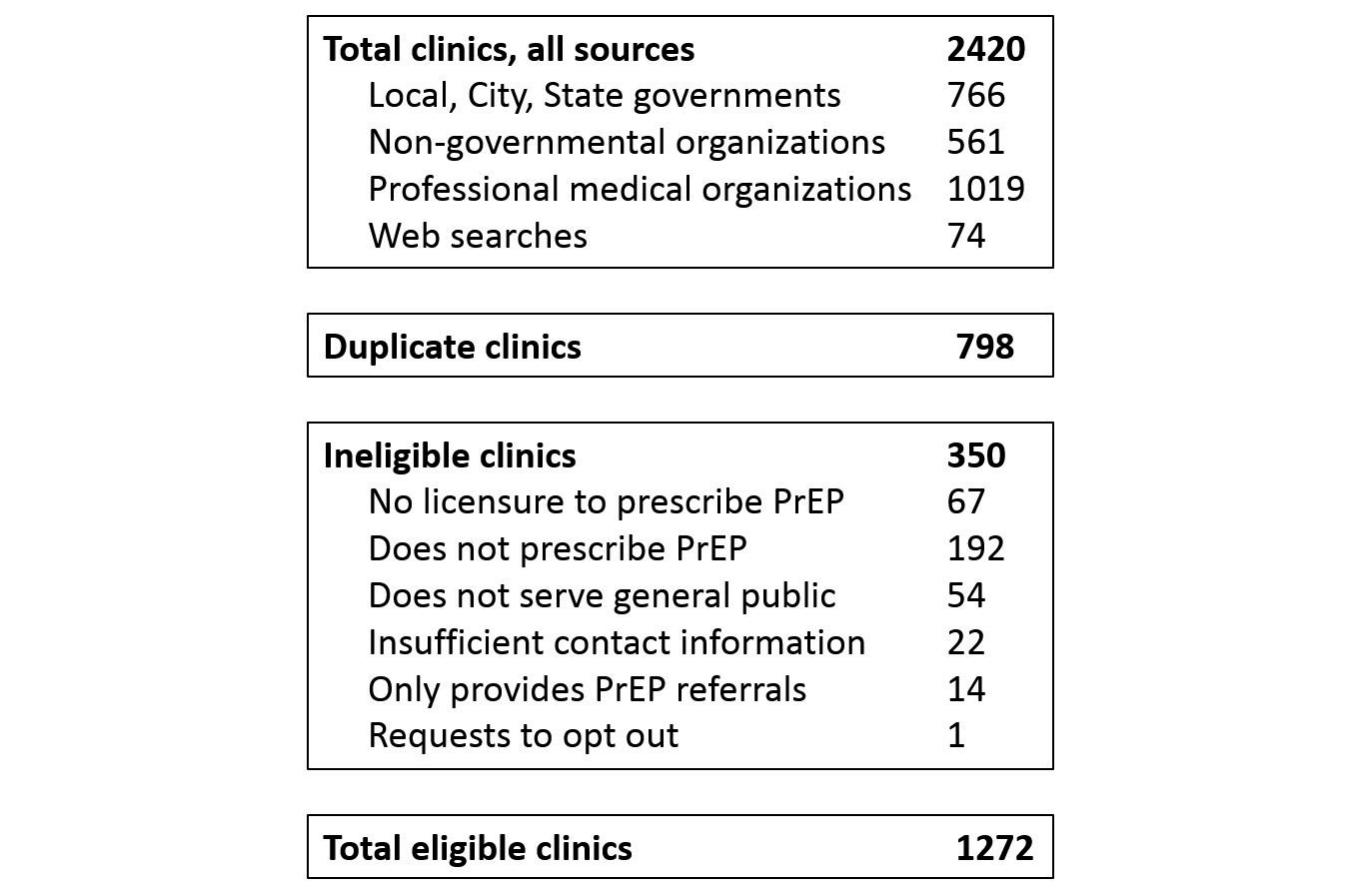
Data sources for clinics populating the PrEP Locator database at project release, September 2016.

From this group of 2420, we determined 1272 clinics to be eligible for inclusion, comprising the final set of clinics publicly listed in the database at the release of preplocator.org (Figure 1). We removed one-third of clinics (798/2420) because these were duplicate entries. Of the remaining unique clinics, 22% (350/1622) were ineligible. Common reasons for ineligibility included self-report as not prescribing PrEP (55%, or 192/350), not having current medical licensure to prescribe PrEP (19%, or 67/350), and not serving members of the general public (eg, student-only clinics) (15%, or 54/350). The final dataset of eligible clinics included PrEP-providing clinics in all 50 states and in Puerto Rico.

In the 6 months following release, preplocator.org and hosting websites had received over 35,000 unique views and over 45,000 views in total. The sites attracted this traffic without a substantial advertising campaign, instead relying on advertisements donated by a geosocial networking app company and by websites hosting the Web tool. Over 300 new clinics had been added to the PrEP Locator database, mostly through webform additions by clinics seeking to join the database. Five websites had initiated hosting of the Web tool by embedding it into their websites, allowing for their site users to have real-time access to the PrEP Locator database. Web tool users include a local health department campaign, a foundation, and community-based organizations.

##### Adding New Clinics to the Database

New entries in the database are added through two mechanisms. In the first, participating state and local health departments are asked to add any clinics newly added to their local databases to the PrEP Locator database at minimum biannually, with a goal of monthly information updates. Updates are communicated through webform entries or through electronic communication. Emory staff update the directory with information for new clinics after appropriate vetting to determine eligibility. Similarly, Emory staff make any corrections needed for previous entries, as the information is received from webform entries. State or local directories willing to participate in more real-time mechanisms to update their information will be accommodated.

The second method for the addition of new clinics to the database is based on crowdsourcing. Both the widget display and the PrEP Locator website have links to a webform (Multimedia Appendix 2) for the addition of new clinics. The database can accommodate requests directly from clinics that wish to join the database and also any clinic suggestions from the public. The webform version presented to those identifying as staff at a clinic includes all major data elements for the database. The webform for those identifying as members of the public not affiliated with a particular clinic only has data categories that would be publicly available, such as address and clinic contact information. For all new clinics, we conduct our standard vetting procedures. When members of the public external to the organization suggest an organization, we conduct standard eligibility assessment and also ensure, through a telephone call or electronic communication, that the organization wishes to opt into the database, and we verify that the organization prescribes PrEP.

New clinics added to the database enter a “holding pen”, a part of the database dedicated to non-publicly listed clinic data to be stored. Emory staff review data in the holding pen for eligibility prior to finalization and public listing of the information in the database. Emory maintains a list of clinics previously determined to be ineligible for the database and checks any newly added data against the ineligible list. Emory staff generally complete determination of clinic eligibility within 2 weeks. Once this is complete, the person or organization responsible for completing the webform entry receives an email describing the eligibility determination.

##### Directory Editing or Removal

Clinics and the public can edit the details of any listing by searching on preplocator.org or the widget for the address of a particular clinic. Clicking on the clinic name or pin will result in display of more detailed information that includes a link labeled “Notify Us of Information Update”. Clinic staff and the public can fill out this information update webform, and their corrections enter an update “holding pen”. Emory staff determine whether the update is accurate and properly sourced within 2 weeks of the information being submitted and send an update determination email to the clinic as well as the member of the public, if applicable. If a clinic or provider wants to remove themselves from the directory, the same steps as above can be followed for the information update webform. Emory maintains records for clinics opting out of the database to ensure that staff do not mistakenly add these clinics back into the database.

#### Data Fields

The database core areas of data collection include contact information, address, PrEP service information, clinic hours, database management information, and system auto-generated variables (Table 1). The database contains a single entry per unique physical address, rather than including multiple entries representing each medical provider at a shared practice or clinic. This approach mirrors that of previous databases, such as the CDC National Prevention Information Network [27]. Having only a single entry per address is beneficial for a location-based interface, as it simplifies the user’s view. Additionally, it increases the long-term feasibility of keeping the database up to date so that provider changes within a clinic do not need tracking over time. For implementation purposes, a single entry is made, with the *title* variable being the clinic or organization’s name. Practitioners without affiliation with a named clinic or practicing outside of a named clinic, at a separate address, are permitted to use their name as the *title* variable in the database.

Address information is collected to allow for geolocation of each clinic, including street information (*streetAddress*), city and/or county (*locality*), zip code (*postalCode*), and state (*region*). Contact information, collected to allow the public to communicate with the facility, includes the clinic telephone number (*telephone*), email address (*clinicEmail*), and website (*link*).

Based on guidance from the project’s steering committee, the database includes information on four key services: (1) *Spanish* language: “Do you have Spanish-speaking clinic staff?” (2) services for the *uninsured*: “Do you offer PrEP if a patient does not have insurance?” (3) PrEP *coordinator*: “Do you have a PrEP coordinator in your clinic or practice?” and (4) PrEP *navigation*: “Does your practice help patients navigate paying for PrEP (eg, reviewing insurance, identifying coverage and deductible gaps, and assisting with enrollment in appropriate programs)?” Each of these service offerings is assessed based on clinic self-report, with binomial (Yes or No) responses. If a clinic has a PrEP coordinator, additional questions are asked to collect contact information, and this information becomes an object in the database.

Additional areas of data include clinic hours (captured as a single database object), data entry management variables, and system-generated variables. Data entry management variables are used to manage the system and include the *status* of the entry (active, pending, or excluded (ineligible)), whether an entry has been *vetted* by Emory staff for proper licensure to prescribe PrEP, and *notes* regarding eligibility determination for each clinic. Certain read-only, system-generated variables are automatically generated by the system based on predefined rules: unique *id* number, latitude (*lat*) and longitude (*long*) of each clinic based on the clinic’s address and a Google application programming interface (API) callout, and date and time variables to indicate when each clinic entry was *created* and/or most recently *updated*.

**Table 1:** PrEP Locator database system domains, variables, variable definitions, and variable examples.

Domain and variable name		Variable definition	Variable example
**Contact information**			
	Title^a^	Name of the clinic or provider	Empowerment Resource Center
	Telephone^a^	Clinic telephone number object, containing a number that must be 10 digits or empty and an extension field without an enforced format	404-999-9999
	Telephone2	Second clinic telephone number object	404-999-5555
	ClinicEmail	Email address of the clinic, which must be a valid email address	info@preplocator.org
	Link	Website of the clinic, which must be a valid URL beginning with http:// or https://	https://www.preplocator.org
**Address**			
	StreetAddress^a^	Street address	1518 Clifton Rd.
	Locality^a^	City and/or county	Decatur
	Region^a^	State	GA
	PostalCode^a^	5-digit zip code	30322
**PrEP service information**			
	Spanish	Whether the clinic offers services in Spanish; false by default	False
	Uninsured	Whether the clinic accepts uninsured patients; false by default	True
	Navigation	Whether the clinic offers PrEP navigation services; false by default	True
	Coordinator	PrEP coordinator for the clinic, an object containing the clinic coordinator’s information, with name, email address, and telephone number fields	Jane Doe, janedoe@healthcare.com, 404-727-9999
**Clinic hours**			
	Schedule	Schedule for the clinic, an object containing the hours of operation, with objects for each day of the week; each day’s object contains status (open, closed, or empty), openTime, and closeTime	Monday, open 8:00AM to 5:00PM
**Entry management**			
	Status^a^	Active, pending, or excluded, as determined by Emory staff	Active
	Vetted^a^	State licensure verification by Emory staff	True
	Eligible	Rationale for eligibility determination	Does not prescribe PrEP
	Notes	Contact notes for each clinic, including information such as the number of contact attempts and the results of contact	Contacted by email and telephone 5 times.
**System-generated variables**			
	Id	ID of the clinic, auto-incremented from 1	1428
	Lat	Latitude in decimal format, generated from a Google API using the clinic address	33.798258
	Long	Longitude in decimal format, generated from a Google API using the clinic address	-84.323467
	Created	RFC 3339 timestamp for when the clinic was added to the database	2016-04-12T23:20:50.52Z
	Updated	RFC 3339 timestamp for when the clinic was added to the database	2016-04-12T23:20:50.52Z

^a^Indicates a required variable; an entry cannot be processed without this information.

For all non-required variables, information may not be available for each clinic for all data fields. In the case of incomplete information, each variable is set to empty.

#### Data Validation

Emory staff conduct data validation checks to ensure the accuracy of the information in the directory. Website links are checked to ensure that they link to active websites. For clinics that list a Facebook page as their primary website, Emory staff check to ensure that the Facebook page has been updated in the last 6 months. If Facebook websites have not been recently updated or if we are unable to locate an active and functioning link for a standard website, we set the *link* variable to empty.

We developed a function for duplicate identification via an algorithm that uses distance, address, and name similarity. Emory staff manually review potential duplicates identified through the algorithm to determine whether a follow-up call with the clinic(s) is merited for clarification. Staff then determine whether no change is needed, a duplicate entry needs to be determined to be ineligible, or fields from an entry need to be edited for one or both clinics.

#### PrEP Locator Access

A location-based search widget that can be easily embedded into any website allows organizations or individuals to place the PrEP Locator on their website and have real-time access to the PrEP Locator’s database using an API. There are no restrictions on use of the PrEP Locator widget. Information necessary to embed the tool is available at https://preplocator.org/publicly-available-tools/. The widget can auto-detect user location, via permission to use the device’s location option, or users can enter their address or zip code. Using a Google API, the widget lists PrEP clinics by nearest location. Implementation of the widget can be seen at https://preplocator.org/ and also is hosted at https://www.greaterthan.org/get-prep/. The PrEP Locator currently has several features, including search filtering options for the uninsured and navigator variables. It also uses an algorithm to automatically expand the search area if there are no PrEP clinics or providers proximal to a given search location. Therefore, if there are no clinics within 25 miles of the user-determined location, the widget will automatically expand the search area outward, with a maximum search area of 100 miles. The widget is compatible with desktop (screenshot in Multimedia Appendix 3) and mobile (screenshot in Multimedia Appendix 4) devices.

#### Database Information

##### Database Specifications

The database is a RESTful API service programmed in Go and hosted on Amazon Web Services (AWS EBS). The database uses Amazon Relational Database (AWS RDS) with the open-source, object-relational database PostgreSQL engine. Locations can be queried individually (by ID) or with search parameters (including geography) and can be added (POST), updated (PUT), or deleted (DELETE). Authentication is done via basic HTTP authentication over SSL with per-client credentials that will be provided. Clients are listed, added, or deleted, and clients have read, write, and/or admin permissions.

##### Database Compatibility

In order to facilitate ease of use and data access, the data fields of the PrEP Locator have name and format conventions that are in line with existing databases, such as the National Prevention Information Network presented by the CDC. Additionally, staff will continue to seek to provide functionality for other partners wishing to access the data.

##### Database Management

For security purposes, database access will be role based and limited to Emory staff working on this project, who have each completed appropriate training regarding the data system and its management and operation. Rights-based permissions will be used to allow database updating and changes.

Records for all clinics, including those deemed ineligible, will be maintained in the database to facilitate screening of clinics suggested for inclusion. The records of clinics determined to be ineligible will be kept internal to Emory; their records will not be shared with or viewable by external partners unless the listings maintained by database partners, such as state health departments, need to be updated.

##### Database Updating

Above, we describe our system for regular information updates from state and health department databases and options for crowdsourced, clinic staff, or public updating of individual clinic entries. To further maintain the accuracy of the information in the directory, an email will be sent biannually to each clinic that provided a contact email address. The update email asks clinic staff to verify that the information in the directory is correct. Any edits are vetted through the process described above. For clinics or providers without electronic contact information, we will attempt to make calls annually to ensure that information in the database is current.

##### Collaborations

The Collaborators Board for the project is responsible for making recommendations for addressing the challenges of this project throughout its development as well as for long-term strategies for the successful distribution and utilization of the PrEP Locator database. The Collaborators Board includes staff representatives from the University of California – San Francisco, the National Alliance of State and Territorial AIDS Directors (NASTAD), Gilead Sciences, and the Greater Than AIDS (represented by the Kaiser Family Foundation). The External Advisory Committee for the project is responsible for providing guidance regarding the development of the PrEP Locator. The desired result of this input is to maximize the acceptability of the resource to clinics, current directory owners, and end users. It includes participants affiliated with the CDC and with AIDS.gov (see Multimedia Appendix 5 for the full list of Collaborators Board and External Advisory Committee members as well as for the charters for each group).

##### Legal

Attorneys in the Emory legal department guided the process of addressing the copyright ownership issues related to the data, the development of the database, and the distribution of the widget. The Emory legal team also suggested or reviewed language regarding disclaimers of use. Additionally, the Emory legal department developed the Terms of Use for the contents of the directory, seen in Multimedia Appendix 6, and informed our development of a privacy policy, seen in Multimedia Appendix 7.

## Discussion

Patients often need to locate a clinician, such as when they are seeking a new service or seeking services after moving to a new geographic area. Clinic and clinician directories facilitate appropriate identification of clinicians for a broad array of health services, such as services for traumatic stress [28]; transgender or LGBT health [29]; obesity [30]; pediatric autism spectrum disorder [31]; or HIV, sexually transmitted diseases, or hepatitis [32,33]. As new treatment and prevention modalities are developed, there will be a corresponding need to develop new clinic directories. Early choices in the development of service directories have a long-lasting impact due to the labor-intensive nature of conducting the initial database population. The PrEP Locator protocol may inform decisions in the development of future service directories.

Key informant interviews and advisory board input shaped a number of important decisions regarding database development and dissemination, particularly by emphasizing project feasibility and end-user perspectives. The use of clinics as the unit of data, instead of individual clinicians, was key to the project’s feasibility because the total number of data lines requiring manual review, 2420, would have been substantially larger if individual clinicians were enumerated. Similarly, suggestions to reduce eligibility criteria to a few key variables and to minimize other areas of data collection to those most relevant to the patient end users facilitated streamlined creation and future management of the database.

Our use of a patient-centered approach served as a useful framework to inform many of the directory development decisions. We collected data on PrEP financial navigation services and on clinics that serve patients without insurance because our advisory boards identified these as important and because making the information publicly available could remove access barriers. Concepts regarding our targeted user-interface design informed our database design. As anticipated, the majority of Web traffic came from mobile phones. For mobile devices with smaller screens, a simplified interface was required (Multimedia Appendix 4), with active links that could facilitate use. Data should be collected to allow for a mobile-relevant interface; a tap on the telephone number, email address, or mailing address of the clinic should lead to opening of the appropriate functionality (dialer, mail, or navigation map). Similarly, database variables should be questions with binomial or categorical answers as often as possible, as this answer type easily translates into check-box search filters.

Management of data entry and verification is essential, so database construction requires building back-end functionality. The public can suggest new data, consisting of either corrections of existing listings or suggestions for new listings, through webforms, and these data must be managed. Including a “holding pen” where data are stored prior to public availability requires additional staff effort to maintain but ensures that the public can contribute to the database and that staff can vet data. Building an efficient back-end (not publicly facing) system for data entry and management provides substantial benefit, allowing for efficient and accurate data entry by staff, facilitating adherence to directory protocols, and aiding project oversight and management. Additionally, building a back-end system with appropriate data formatting and data management can serve to facilitate potential future integration with complementary external databases for other services. For instance, adding a PrEP services database to a sexually transmitted infection services database could benefit users by allowing them to search services in a single hub.

Geolocated directories for service provision have the potential to assist patients in seeking needed health services. Such directories have the potential to maximize the ease of patient access by incorporating electronic and Web-based design components as well as appropriate database structure and design to support the patient-facing interface. Gathering information from key stakeholders and those with database development experience provides substantial utility. This protocol may serve as a starting point for future directory development.

## Appendix

Multimedia Appendix 1 Script for calls to clinics/ health centers/providers that prescribe PrEP.

Multimedia Appendix 2 Clinic addition webform.

Multimedia Appendix 3 Desktop widget interface.

Multimedia Appendix 4 Mobile widget interface screenshots.

Multimedia Appendix 5 PReP locator.

Multimedia Appendix 6 Terms of use.

Multimedia Appendix 7 Privacy policy.

## Abbreviations

API: application programming interface
CDC: Centers for Disease Control and Prevention
MSM: men who have sex with men
NASTAD: National Alliance of State and Territorial AIDS Directors
PrEP: pre-exposure prophylaxis
