# Supplementary material for: Developing a Web-Based Geolocated Directory of HIV Pre-Exposure Prophylaxis-Providing Clinics: The PrEP Locator Protocol and Operating Procedures
Source: JMIR Public Health Surveill. 2017 Sep 6;3(3):e58. doi: 10.2196/publichealth.7902 (PMC5607436; doi:10.2196/publichealth.7902)
Supplement: Multimedia Appendix 1 [file publichealth_v3i3e58_app1.pdf]

Multimedia Appendix 1: Script for calls to clinics/ health centers/providers that prescribe PrEP

Items to note before calling:

This call is to confirm the data we have and get information for data fields that are blank. It's not to ask permission to include them in the database.

Be cognizant of time zones. Don't try calling the West Coast at 10 am EST.

Can call clinics in other times zones if a time zone is in the lunch hour, typically noon until 1:00 pm.

When you're in a phone tree and have options for transferring, choose the "appointments" extension. That seems to be the most helpful. Nurses are also helpful but are busy seeing patients during the day.

Script

Hi, I'm calling to find out if you provide PrEP? **OR** Do you prescribe PrEP in your clinic?
[If they don't know what is it say] PrEP is pre-exposure prophylaxis to prevent HIV.
Say "great" or "awesome" if they do provide it. Regardless, sound positive. If they say they don't currently prescribe PrEP but want to/ plan to start, please ask them the date when they will begin prescribing. A follow-up call can be made to make sure they've started.

My name is XXXXX and I work at Emory University in Atlanta and we have a grant to put together a national directory of PrEP providers.

I was wondering if I could ask you or anyone else on your staff a couple questions about PrEP and its policies at your clinic. [If they ask what kind of questions say general info, payment and insurance questions] **If they say they cannot answer ask for someone who can**

Do you prescribe PrEP for patients that don't have insurance? **OR** Do you take patients that need PrEP if they don't have insurance?

Do you have someone on staff that is PrEP navigator, meaning they can help a patient get enrolled in payment assistance program that can help them pay for PrEP, or help pay for the clinic visits? [** I only say this next part if the person seems confused**]For example, Gilead has a drug assistance program to help people pay for PrEP because it is $1300 a month.

Do you have someone in the clinic that is what we call a "PrEP coordinator", someone that can answer patients' questions about PrEP? If so, can I have their name and phone number to include in the directory?

Do you have any clinic staff that speak Spanish?

Is your address still XXXXX [give full address and let them confirm]

And the name of your clinic is still XXXX?

Is this the best number for someone to call if they are interested in PrEP? **OR** Is there a more direct phone number for someone to call if they are interested in PrEP?

What are your hours?

Can you give me an email address for a clinic staff person who can receive two emails per year that are sent to make sure that the clinic information we have in the directory is still correct? The mail will NOT be shared or made public. It will only be used to confirm the current clinic information. **OR** Can you give me the email for your office manager? [Can then explain why it's requested. A lot of times I have found people do not know one, still ask the question anyway]

Thank you so much for your time! I really appreciate it.
