# Supplementary material for: Developing a Web-Based Geolocated Directory of HIV Pre-Exposure Prophylaxis-Providing Clinics: The PrEP Locator Protocol and Operating Procedures
Source: JMIR Public Health Surveill. 2017 Sep 6;3(3):e58. doi: 10.2196/publichealth.7902 (PMC5607436; doi:10.2196/publichealth.7902)
Supplement: Multimedia Appendix 2 [file publichealth_v3i3e58_app2.pdf]

Multimedia Appendix 2: Clinic addition webform

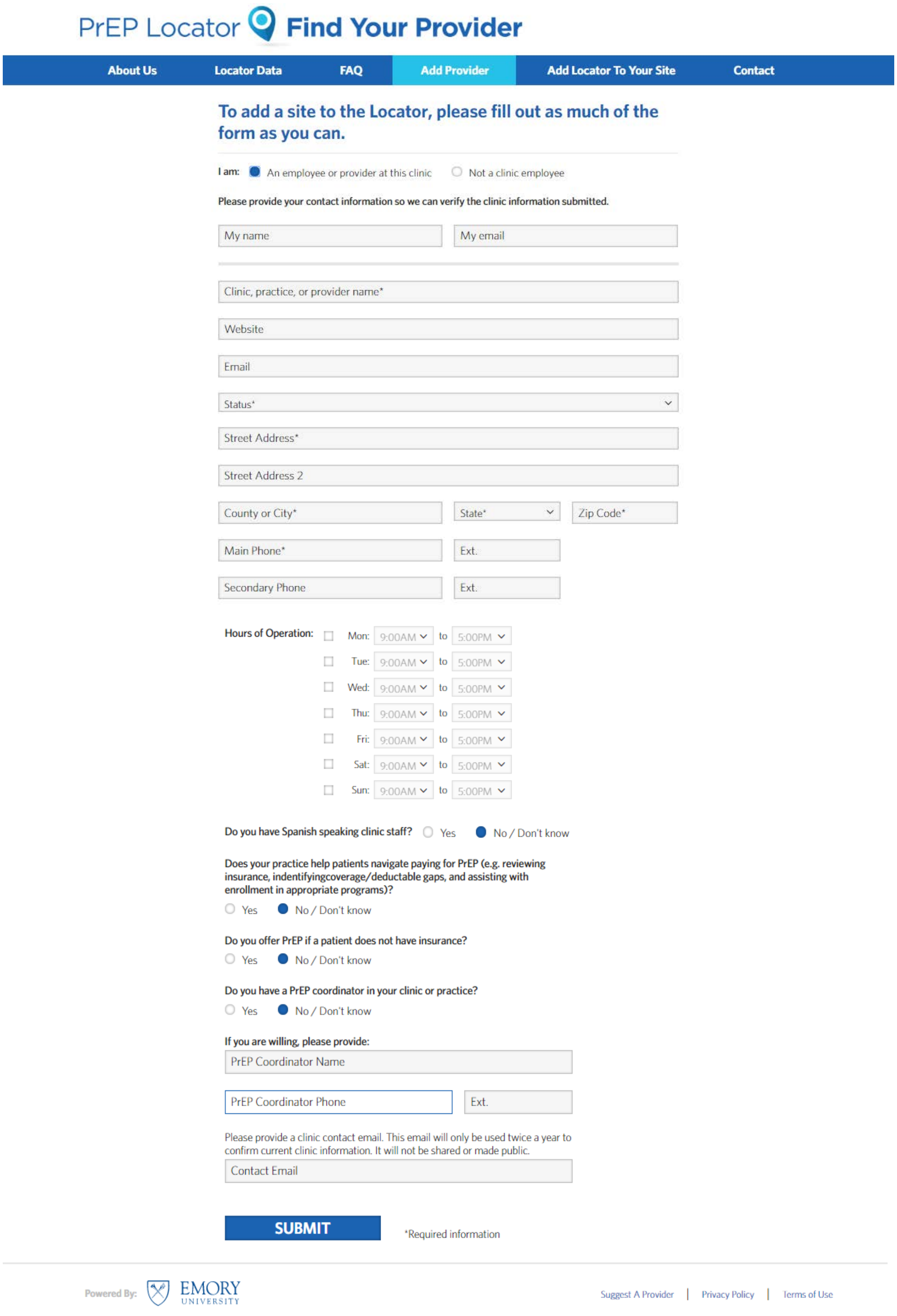

PrEP Locator Find Your Provider

About Us | Locator Data | FAQ | Add Provider | Add Locator To Your Site | Contact

To add a site to the Locator, please fill out as much of the form as you can.

I am: ● An employee or provider at this clinic ○ Not a clinic employee

Please provide your contact information so we can verify the clinic information submitted.

My name

My email

Clinic, practice, or provider name*

Website

Email

Status*

Street Address*

Street Address 2

County or City*

State*

Zip Code*

Main Phone*

Ext.

Secondary Phone

Ext.

Hours of Operation:

Mon: 9:00AM to 5:00PM

Tue: 9:00AM to 5:00PM

Wed: 9:00AM to 5:00PM

Thu: 9:00AM to 5:00PM

Fri: 9:00AM to 5:00PM

Sat: 9:00AM to 5:00PM

Sun: 9:00AM to 5:00PM

Do you have Spanish speaking clinic staff? ○ Yes ● No / Don't know

Does your practice help patients navigate paying for PrEP (e.g. reviewing insurance, indentifyingcoverage/deductable gaps, and assisting with enrollment in appropriate programs)?

○ Yes ● No / Don't know

Do you offer PrEP if a patient does not have insurance?

○ Yes ● No / Don't know

Do you have a PrEP coordinator in your clinic or practice?

○ Yes ● No / Don't know

If you are willing, please provide:

PrEP Coordinator Name

PrEP Coordinator Phone

Ext.

Please provide a clinic contact email. This email will only be used twice a year to confirm current clinic information. It will not be shared or made public.

Contact Email

SUBMIT

*Required information

Powered By: EMORY UNIVERSITY

Suggest A Provider | Privacy Policy | Terms of Use
