# Supplementary figures and images for: Developing a Web-Based Geolocated Directory of HIV Pre-Exposure Prophylaxis-Providing Clinics: The PrEP Locator Protocol and Operating Procedures
Source: JMIR Public Health Surveill. 2017 Sep 6;3(3):e58. doi: 10.2196/publichealth.7902 (PMC5607436; doi:10.2196/publichealth.7902)

## Multimedia Appendix 4: Mobile widget interface screenshots

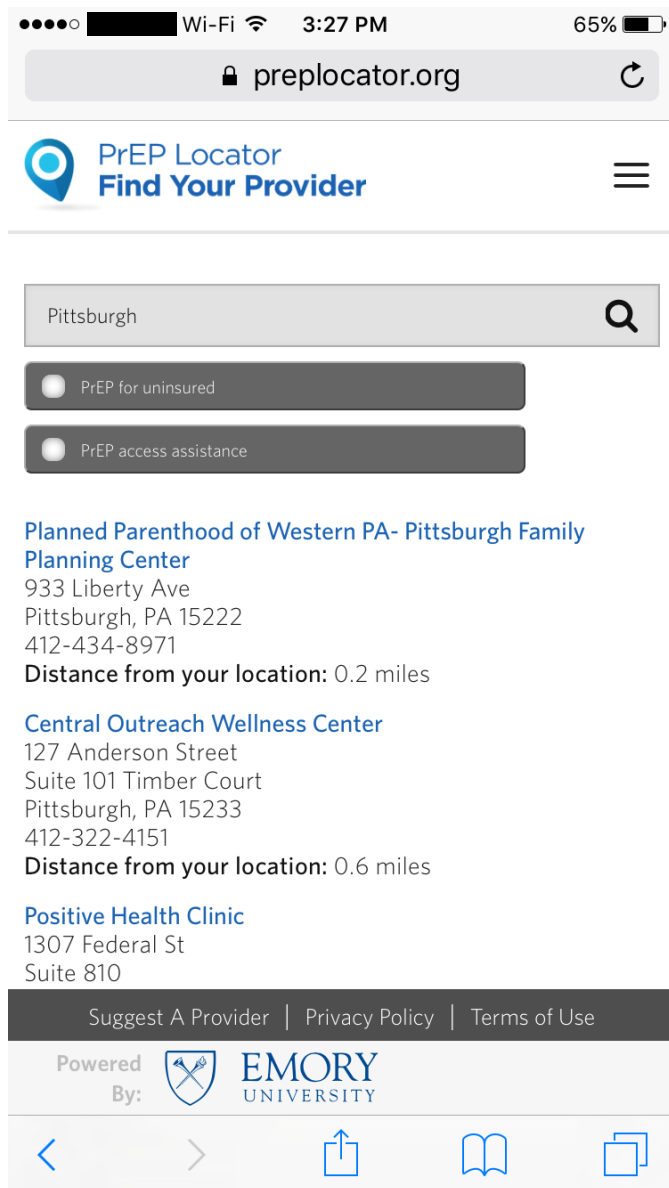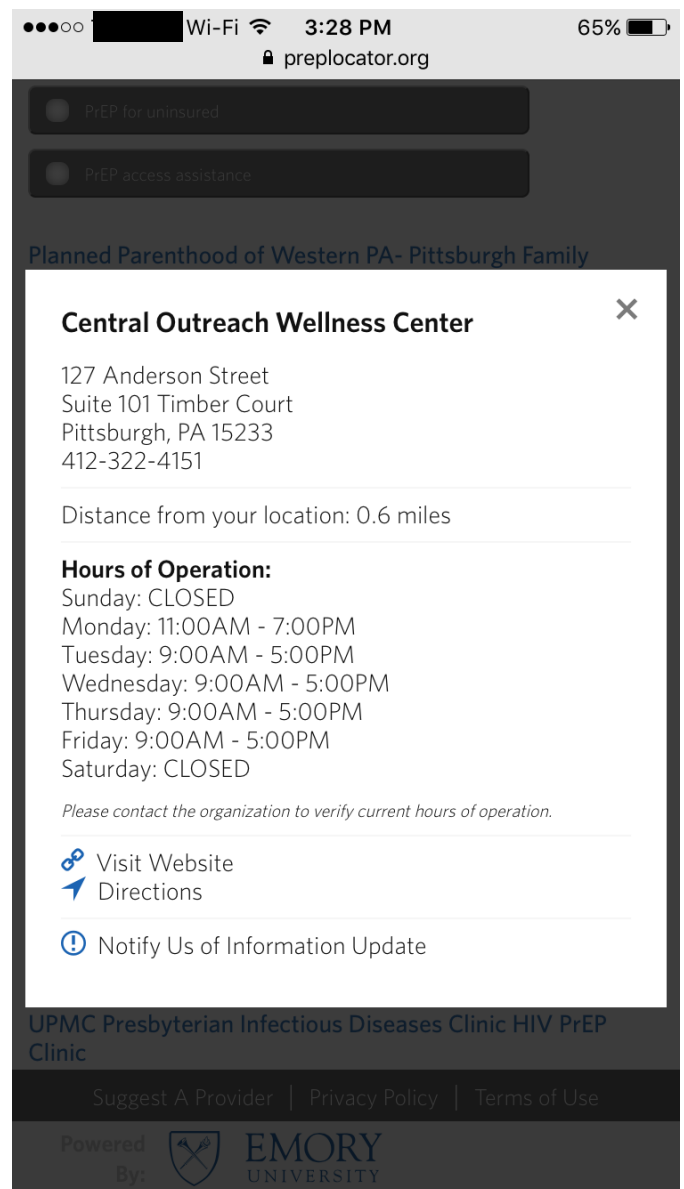

Supplement: Multimedia Appendix 4 [file publichealth_v3i3e58_app4.pdf]
