# Supplementary material for: Developing a Web-Based Geolocated Directory of HIV Pre-Exposure Prophylaxis-Providing Clinics: The PrEP Locator Protocol and Operating Procedures
Source: JMIR Public Health Surveill. 2017 Sep 6;3(3):e58. doi: 10.2196/publichealth.7902 (PMC5607436; doi:10.2196/publichealth.7902)
Supplement: Multimedia Appendix 5 [file publichealth_v3i3e58_app5.pdf]

Multimedia Appendix 6: Terms of Use

PrEP Locator Terms of Use

The information contained in this PrEP Locator database may not be used in any way for commercial purposes. The information in this PrEP Locator database about each individual clinic, provider, hospital or health care site can change. It may also be incomplete or inaccurate. It is recommended that you contact a site directly to verify the information presented here.

"NO ENDORSEMENTS; GUARANTEES OR WARRANTIES. EXCLUSION OF LIABILITY. The information and content contained in the PrEP Locator database (the "Locator") contains names and contact information of certain physicians or other healthcare providers, product providers, or medical products that was compiled using publicly available lists and information. Emory cannot guarantee the accuracy of the Locator. Emory University and Emory Healthcare, Inc. ("Emory") does not endorse any particular physician, healthcare provider, product provider, or medical product or any information in the Locator. The Locator is created for information purposes only and is independent of any provider, device or drug manufacturer.

EMORY IS NOT (I) MAKING ANY REPRESENTATIONS OR WARRANTIES REGARDING ANY PROVIDER, DRUG OR DEVICE, OR (II) ASSUMING ANY RESPONSIBILITY FOR ANY USE OR MISUSE OF THE LOCATOR.

IN NO EVENT WILL EMORY ASSUME ANY RESPONSIBILITY OR LIABILITY FOR ANY USE OR MISUSE OF THE INFORMATION OR CONTENT PROVIDED ON OR ACCESSED THROUGH THE LOCATOR. IN NO EVENT WILL EMORY OR ITS PARENT OR AFFILIATE COMPANIES BE LIABLE TO YOU OR ANYONE ELSE FOR ANY ADVICE OR TREATMENT PROVIDED, DECISION MADE OR ACTION TAKEN BY A PROVIDER, YOU OR ANYONE ELSE IN RELIANCE UPON THE INFORMATION OR CONTENT CONTAINED ON OR ACCESSED THROUGH THE LOCATOR."
