# Supplementary material for: Developing a Web-Based Geolocated Directory of HIV Pre-Exposure Prophylaxis-Providing Clinics: The PrEP Locator Protocol and Operating Procedures
Source: JMIR Public Health Surveill. 2017 Sep 6;3(3):e58. doi: 10.2196/publichealth.7902 (PMC5607436; doi:10.2196/publichealth.7902)
Supplement: Multimedia Appendix 7 [file publichealth_v3i3e58_app7.pdf]

Multimedia Appendix 7: Privacy Policy

PrEP Locator Privacy Policy

This Website Privacy Statement explains how PrEP Locator handles the information that you provide or that is otherwise collected. This Website Privacy Statement does not apply to apply to Google Maps, which provides the maps and directions for PrEP Locator. This Website Privacy Statement also does not apply to any websites that may choose to display the PrEP Locator widget or use data from the PrEP Locator Database. You are encouraged to review the terms of the privacy policy for Google Maps and for any websites using the PrEP Locator widget or otherwise displaying data from the PrEP Locator database.

How does PrEP Locator collect information online?

PrEP Locator may collect information in two ways:

Passive Collection of Unidentifiable Information: PrEP Locator may collect information about your visits to the Locator website, without you actively submitting such information. Unidentifiable information may be collected using various technologies, such as cookies and web beacons. Cookies are small text files that are transferred to your computer's hard disk by a website. Web beacons (also referred to as GIF files, pixels, or Internet tags) help PrEP Locator recognize a unique cookie on your browser. Your Internet browser automatically transmits to PrEP Locator some of this unidentifiable information, such as the URL of the website you just visited and the browser version your computer is operating. Passive information collection technologies can make your use of PrEP Locator easier by allowing the Locator to provide better service, customize based on consumer preferences, compile statistics, analyze trends, and otherwise administer and improve the Locator. Certain features of PrEP Locator may not work without use of passive information collection technologies. Information collected by these technologies cannot be used to identify you without additional identifiable information and the Locator will not match additional identifiable information with information collected through the use of such tracking technologies.

Personal Information You Submit: PrEP Locator collects Personal Information in three areas. The first is on the online form for suggesting a provider or clinic to add to the Locator. The second is the online form to update a provider or clinic's information as shown in the PrEP Locator. Any names and/or emails voluntarily provided will not be made public, shown in the Locator, nor be shared or used for any purpose outside of the Locator unless we gain provider assent to be added to the Locator database. The third is the “Contact us” section, where users may provide feedback or ask a question. To protect your privacy, you should not provide PrEP Locator with any information that is not specifically requested.

You may choose to opt-out of the PrEP Locator at any time. Your request to opt-out will be honored after we verify the request.
